# Exploring Knowledge and Attitudes about Vitamin D among Adults in Saudi Arabia: A Qualitative Study

**DOI:** 10.3390/healthcare5040076

**Published:** 2017-10-16

**Authors:** Najlaa Aljefree, Patricia Lee, Faruk Ahmed

**Affiliations:** 1 Public Health, School of Medicine and Menzies Health Institute Queensland, Griffith University, Gold Coast campus, Southport QLD 4222, Australia; patricia.lee@griffith.edu.au (P.L.); f.ahmed@griffith.edu.au (F.A.); 2 Department of Medical Research, China Medical University Hospital, China Medical University, Taichung City 404, Taiwan

**Keywords:** vitamin D deficiency, knowledge, attitudes, qualitative, sun exposure, vitamin D supplements, Saudi Arabia

## Abstract

Vitamin D deficiency is widespread in Saudi Arabia. The aim of this study was to explore participants’ knowledge about vitamin D and attitudes toward sun exposure. The study also aimed to explore the social and cultural factors that might potentially contribute to vitamin D deficiency in Saudi Arabia. Face-to-face interviews were carried out in the cities of Jeddah and Makkah between May and October 2015. The interview questions were semi-structured, and the data was analyzed using thematic analysis. Study participants showed a reasonable level of knowledge in different areas about vitamin D, including the effect of vitamin D deficiency on bone health and exposure to sunlight as the main source of vitamin D. Participants were also knowledgeable about vitamin D supplements as another source of this vitamin. Nevertheless, there was a shortage of knowledge in relation to dietary sources of vitamin D. In respect to attitudes toward sun exposure, some participants had positive attitudes toward sunlight and were willing to expose themselves to sunlight, but it was restricted to the early morning or late afternoon to avoid the heat. These participants who liked exposure to sunlight were largely exposing only their faces and hands to sunlight. Other participants had negative attitudes toward sun exposure and were avoiding sunlight. Moreover, the study participants identified several barriers to sun exposure, including hot climate, living in high-rise buildings, limited public areas allowing outdoor activities, lifestyle issues such as physical inactivity, and some religious concerns such as wearing the hijab. The study results also demonstrate that females were more enthusiastic about taking actions to improve their vitamin D status in comparison with males. Recommendations for health education interventions that increase awareness about vitamin D sources, especially food sources, are made. Also, educational interventions should focus on increasing awareness about the sufficient time of the day and duration for sun exposure to improve vitamin D status and the importance of the intake of vitamin D supplements as an affordable source to improve vitamin D status. Increasing males’ awareness of the benefits of vitamin D is important to encourage them to adopt behaviors to improve vitamin D.

## 1. Introduction

Vitamin D deficiency is highly prevalent in sunny countries such as Saudi Arabia. Previous studies within the country indicated a high rate of vitamin D deficiency among both males and females [1,2]. A recent national survey reported high rates of vitamin D deficiency: 40% in males and 60% in females in Saudi Arabia [3]. Vitamin D deficiency has historically been known to increase the risk of some musculoskeletal disorders, such as osteoporosis [4]. Furthermore, several studies have shown an association between vitamin D deficiency and other major chronic diseases such as cancer, diabetes, and cardiovascular disease (CVD), worldwide [5,6,7]. Recently, we reported an independent association between vitamin D deficiency (25(OH) D < 20 ng/mL) and coronary heart disease (CHD) among adults in Saudi Arabia (OR: 6.5, 95% CI: 2.7–15) (*p* ≤ 0.001) [8], as well as an association between vitamin D deficiency and diabetes among adults with CHD in the Kingdom (OR: 2.9, 95% CI: 1.02–8.5) (*p* = 0.04) [9]. These findings suggest that prevention of vitamin D deficiency is vital to reducing the risk of CHD and its associated risk factors in Saudi Arabia.

Several studies have focused on the biological factors that cause vitamin D deficiency around the world; however, few studies have examined the effects of cultural and lifestyle behaviors, knowledge and beliefs that influence vitamin D status [10,11,12]. Recently, we conducted a study using a quantitative approach to examine knowledge and attitudes about, and behaviors toward, vitamin D in subjects with and without CHD in Saudi Arabia [13]. The results showed that low levels of knowledge about vitamin D and low consumption of vitamin supplements, including multivitamins and vitamin D supplements, were associated with vitamin D deficiency [13]. Hence, the present study was designed to better understand the underlying reasons for the differences in knowledge about vitamin D as well as to explore in more depth behaviors related to vitamin D in Saudi Arabia. The present study also identified the cultural and social factors that might influence vitamin D related knowledge and attitudes in Saudi Arabia. The findings of this study provide additional information that can be useful for developing targeted health promotion and educational interventions concerning vitamin D deficiency in Saudi Arabia. Therefore, the aims of this qualitative study were to (1) explore participants’ knowledge and their sources of information about vitamin D; (2) explore participants’ attitudes regarding sun exposure; and (3) explore the social and cultural factors that might potentially contribute to vitamin D deficiency in Saudi Arabia.

## 2. Methods

### 2.1. Study Design

In-depth interviews were conducted between May and October 2015 in three hospitals located in the western region of Saudi Arabia (in the cities of Jeddah and Makkah). The in-depth, face-to-face interview method was chosen because some cultural issues are sensitive and thus are better discussed individually rather than in groups. Moreover, due to cultural traditions, it might be difficult to have a group that included both genders, so this method was considered the most appropriate way to collect data in this setting. The interviews were semi-structured, with prepared open-ended questions that were adapted from the literature [11,14,15]. In this study, the interview questions have been modified to suit the Saudi population (questions in Supplementary Materials Table S1).

### 2.2. Recruitment of Study Subjects

Participants were selected through consultation with the study subjects of the quantitative study of this research project during data collection [8]. They were asked whether they were willing to participate in qualitative interviews. The interviews were conducted by the researcher (NA) and took place in the three hospitals where the quantitative study was undertaken: King Abdullah Medical City (KAMC), King Abdulaziz University (KAU), and Tunsi private hospital. The interviews were recorded and then fully transcribed. Ethical approval was obtained from the Griffith University Human Research Ethics Committee (GU Ref No: MED/59/14/HREC), the KAMC Institutional Review Board (IRB No: 15-194), and the KAU Research Ethics Committee (Reference No. 118-15). Study subjects confirmed their agreement to participate in the interviews by signing informed consent forms.

### 2.3. Data Analysis

All interviews were fully transcribed by the researcher (NA) after listening to the voice recordings several times. Thematic analysis was used to analyze the data by manually identifying codes and themes. The coding process and theme development was accomplished by the first two authors. NA conducted the initial coding and grouped similar codes together. PL checked all the coding and modified several codes and extracted the common themes based on issues raised by the study participants, while confirming all contextual information with NA during the interviews. NA and PL also discussed and verified all the developed themes.

## 3. Results

A total of 22 participants took part in the in-depth face-to-face interviews. The saturation of the data was determined at this point as no more new issues were raised by the last one or two participants. The study participants comprised 11 males and 11 females. The majority of study participants were 49 years old and above, married, living in urban areas, Saudis, employees, and well-educated. The socio-demographic characteristics of study participants are shown in Table 1.

Each interview lasted approximately 25–35 min. During the data analysis, four broad themes were revealed: (1) knowledge and information sources about vitamin D; (2) attitudes regarding exposure to sunlight; (3) reasons preventing people from getting sufficient sun exposure; and (4) gender differences regarding taking actions against vitamin D deficiency.

### 3.1. Knowledge and Information Sources about Vitamin D

#### 3.1.1. General Understanding of Vitamin D Deficiency

In the beginning, participants were asked to simply describe what they knew about vitamin D. The purpose of this question was to clarify whether the participants had sufficient knowledge about vitamin D and whether this information was valid. In response to this question, several study participants stated:

Vitamin D is important for bone health; exposure to sunlight is the main source of vitamin D.

In addition, some participants mentioned vitamin D supplements as another source of vitamin D besides sun exposure. A few participants also pointed out food rich in vitamin D as a source of vitamin D.

*I think taking vitamin D supplements is vital to increase vitamin D levels*.

(Participant #22, Male)

*The consumption of food rich in vitamin D and vitamin D supplements would help prevent vitamin D deficiency*.

(Participant #4, Female)

A few participants had unclear information about the intake of food rich in vitamin D and vitamin D supplements. One male participant suggested that eating a variety of food might help to improve his vitamin D status. He stated:

*The intake of different types of fruit and vegetables prevents vitamin D deficiency*.

(Participant #16, Male)

Likewise, another female participant believed that taking vitamin D supplements alone is not efficient to improve vitamin D status. She stated:

*We should not depend on supplements and pills to fill our need for vitamin D; we should take vitamin D from its natural sources*.

(Participant #7, Female)

A few participants named other health benefits related to vitamin D: improving eyesight and depression.

*I know that vitamin D deficiency may cause depression*.

(Participant #1, Female), and

I know that vitamin D is very important to bone health and eyesight.

(Participant #1, Male)

However, three participants stated they did not know anything about vitamin D.

#### 3.1.2. Source of Information about Vitamin D

Study participants were also asked to clarify their source of information about vitamin D. They identified the following sources of information about vitamin D: doctors, media, family and friends, and colleges/universities.

##### Information from Doctors

Many participants had learned about vitamin D through private visits to doctors. Most of them said they did a blood test and found that they were vitamin D-deficient. Also, most of them said that doctors advised them to get exposure to sunlight regularly and to take vitamin D supplements:

After I gave birth to my daughter, the doctor asked me to do a blood test. I was vitamin D deficient and he advised me to take vitamin D supplements.

(Participant #1, Female)

*I learned about the risk of vitamin D deficiency from my doctor. After that, I started searching and reading about vitamin D to gain more information*.

(Participant #4, Female)

Moreover, some participants said that they had learned about vitamin D when they were accompanying parents or wives while visiting doctors.

When I was going with my father to the doctor, I heard about the importance of vitamin D.

(Participant #10, Male)

##### Information from the Media

The media was also a source of information about vitamin D, as the participants mentioned television, the internet, newspapers, and magazines as their sources of information as follows:

I have heard about vitamin D via television programs.

(Participants #8 and 19, Males)

I knew about vitamin D through reading newspapers and the internet.

(Participants #18 and 22, Males)

##### Information from Family and Friends or Learned at College/Universities

A number of participants reported hearing about vitamin D from their family or friends or through learning at colleges and universities. Also, there was one female participant who mentioned that she got her information about vitamin D by attending a large health education workshop. She stated that:

*I heard about vitamin D while attending a workshop that aimed to educate women about their health in Jeddah. In the workshop they said Saudi Arabia has a high prevalence of vitamin D deficiency and Saudi women should take vitamin D supplements*.

(Participant #9, Female)

### 3.2. Participants’ Attitudes about Exposure to Sunlight

#### 3.2.1. Feelings about Sun Exposure

Participants were asked to state how they feel about exposure to sunlight. The purpose of this question was to know whether the participants liked or disliked exposing themselves to sunlight, which is the key source of vitamin D. Some participants expressed positive attitudes toward sun exposure, such as:

I like to expose myself to sunlight and never tried to avoid it.

(Participants #9 and 12, Females)

Although many participants stated that they like sun exposure, they had a preference for sun exposure under certain various conditions. For example, in consideration of high temperature and potentially harmful UV rays in midday, many participants mentioned:

I prefer to expose myself to sunlight only during late afternoon.

(Participant #10, Male)

I like to expose myself to sunlight sometimes, but I tend to avoid the strong, hot sunlight.

(Participants #3, 4, and 20, Females)

Due to some cultural concerns, a few female participants stated that an appropriate place was required for them to uncover their bodies in order to have sufficient sun exposure. They stated:

I like to expose myself to sunlight but I cannot go out, I only expose myself at home via windows.

(Participant #11)

I want to expose myself to sunlight and like sun exposure, but only in an appropriate place where I can take my hijab off.

(Participant #2)

However, some other participants had negative attitudes toward sun exposure, such as:

I do not like sunlight. I rarely get exposed to sun.

(Participants #21, Female; and #8, 15 and 19 Males)

I avoid sunlight because it is too hot; I usually go out for a walk at night.

(Participant #13, Male)

#### 3.2.2. Preferable Time and Duration of Sun Exposure for People Who Do Not Mind Exposure to Sunlight

Some participants mentioned that they do not expose themselves to sunlight during the summer because of the strong heat that might reach 50 °C, especially in the western region cities, such as Jeddah and Makkah.

I usually go out at night but not during the day especially in summer.

(Participant #4, Female)

Moreover, many participants said they prefer early morning (between 6 a.m. and 10 a.m.) or late afternoon to expose themselves to sunlight, to avoid harmful UV rays during the midday, as we mentioned previously:

*I expose myself to sunlight usually during the early morning between 6 a.m. and 7a.m. for less than half an hour*.

(Participant #1, Female)

These participants, however, said that they only exposed their face and hands to sunlight and for approximately 10–30 min. The following quotes illustrate this:

I only expose my face and hands.

(Participant #10, Male, and #9 Female, respectively)

I expose myself to sunlight during the early morning for less than 15 min. Only my face and hands are exposed to sunlight, as I wear the traditional thawb when I go to work every day.

(Participant #18, Male)

Other female participants stated that they prefer the late afternoon (between 4 p.m. and 6 p.m.) to expose themselves to sunlight, as it is a suitable time for them to go to the gym. They also stated that more parts of their body, such as legs and arms, were exposed to sunlight and their exposure to sunlight tended to be for a longer time.

*I expose myself to sunlight when I go to the gym almost three times per week. Usually I expose my legs, arms, and face to sunlight*.

(Participant #2)

On the other hand, only one male participant indicated a higher level of sun exposure due to his work duties as he works outside for several hours on a daily basis. He stated:

*I expose myself for 2–3 h every day, as I work outdoors*.

(Participant #22)

### 3.3. Reasons Discouraging People from Getting Sufficient Sun Exposure

#### 3.3.1. Hot Weather All Year Round

Hot weather, including summer heat and high humidity, was one of the main barriers highlighted by study participants that prevented them from getting adequate sun exposure.

In my opinion, the main reason for vitamin D deficiency is the high temperature and hot weather.

(Participant #22, Male)

The hot weather in Saudi Arabia is one cause of vitamin D deficiency.

(Participants #13 and 17 Males)

*I believe the hot climate in the country is behind vitamin D deficiency.* (Participant #9, Female).

#### 3.3.2. Avoiding Sun Exposure Due to Specific Health Conditions

Some participants mentioned health conditions, such as headache and dizziness, as the main reasons hindering them from getting enough sun exposure. The following quotes indicate that:

*Sun exposure makes me feel dizzy* and *I avoid sunlight; it causes me a headache*.

(Participants #13 and 21, Males)

Another male participant added:

*I have a sun allergy. I cannot expose myself to sunlight*.

(Participant #15)

Similarly, another male participant mentioned sunlight’s effect of causing darker skin as a reason stopping him from exposing himself to sunlight.

*I avoid sunlight; I do not want to have darker skin*.

(Participant #19)

#### 3.3.3. House Designs: Limited Access to an Open Area with Sufficient Sunlight

Many participants reported that house designs were hindering them from getting adequate sun exposure. They mainly admitted that living in units without private balconies was stopping people, especially females, from receiving sunlight. For example, one female stated:

*When I go to my family’s house, I walk freely on their private terrace and expose myself to sunlight; however, I cannot do this at my home because I live in a small unit*.

(Participant #3, Female)

Furthermore, some participants showed a good understanding of the difference between the current and past house architecture in Saudi Arabia. They argued that in the past, houses of most Saudi families had inner spaces that allowed sun rays to enter the house, allowing people to get sufficient sun exposure. In addition, the inside spaces were designed to protect the privacy of the family, as it is an important aspect of Saudi culture. However, the modern design of houses in the country includes completely covered houses or units in high-rise buildings, which clearly do not have this important feature. The following quotes illustrate this:

*Old houses were designed to have uncovered spaces inside the house that allowed sunlight into the house, but now we live in completely covered high-rise buildings*.

(Participant #2, Female)

*House designs in Saudi Arabia are different from the past. We used to have indoor spaces that allowed sunlight to enter the house while remaining private*.

(Participant #8, Male)

#### 3.3.4. Limited Public Areas Enabling Outdoor Activities

Many participants believed that the shortage of outdoor public areas, such as public parks and walking trails, is the main reason for their limited sunlight exposure. Some female participants emphasized that it is as a key problem, especially for females in the Kingdom. They stated:

*We have a limited number of public parks with different facilities. Most entertainment places are indoors and covered because of the hot weather*.

(Participant #3)

*I prefer to join a gym that has an open private area that allows me to be exposed to sunlight*.

(Participant #12)

Likewise, only one male participant pointed out the same issue for young males. He stated:

*There are no outdoor areas for young males to gather that encourage them to expose themselves to sunlight, so they usually go to indoor entertainment centers because of the hot weather*.

(Participant #6)

#### 3.3.5. Lifestyle Issues, Especially Physical Inactivity in Saudi Arabia

Some participants believed that the lifestyle of Saudis might be the reason for the high prevalence of vitamin D deficiency in the country. They pointed out some issues related to having an indoor lifestyle and the lack of physical activity as a barrier to achieving adequate vitamin D serum levels. For example, some male participants said:

*Not practicing exercise, including walking or jogging, is the main reason for vitamin D deficiency*.

(Participants #7 and 15)

Similarly, some participants drew attention to the high dependency on cars for traveling around the city. They stated:

*We do not walk in the street. We usually use cars to go shopping or to work. It might be the reason*.

(Participants #20 and 21, Females)

#### 3.3.6. Cultural and Religious Concerns

Many participants of both genders believed that wearing the hijab might be a factor related to the prevalence of vitamin D deficiency among females in Saudi Arabia. Since the majority of Saudi females wear black abayas as a hijab to cover themselves, participants also stated that the color of the hijab plays a big role in preventing absorption of sufficient sunlight. The following quotes from female participants clarify this:

Wearing abayas might be a reason for vitamin D deficiency among females in Saudi Arabia.

(Participants #4 and 12 Females)

*Because we wear black abayas to cover our bodies outside our homes, we may not be exposed to the sun’s rays during the day*.

(Participant #1, Female)

Some also believed that wearing the hijab can be a big issue but only for females who do not have a proper private place at home that helps them to get sufficient sun exposure.

*Wearing hijab might be the reason for vitamin D deficiency among females, especially those who do not have a private balcony or backyard to help get exposure to sunlight; most families live like this in our units*.

(Participants #12, Female, and #6, Male)

Hijab might be a reason for vitamin D deficiency among Saudi females; however, if they want to prevent themselves from being vitamin D deficient, then it is not an excuse.

(Participant #14, Female)

Nevertheless, other female participants mentioned that they still had a problem with getting exposure to sunlight in a culturally appropriate way even though they have some private uncovered places in their homes. They described the issue as being due to the fact that in Saudi culture and as being a Muslim, women should be hidden from males’ view; thus, they must avoid getting seen by their neighbors, which makes it difficult for them to get sun exposure.

Even though I have a private balcony, the neighbors could see me, so I have to be careful.

(Participant #1, Female)

There were mixed perspectives with regard to the effect of wearing the hijab. Another group of participants had a totally different opinion. They believed that wearing the hijab cannot be a cause of vitamin D deficiency and pointed out other factors, which according to them, are the main reasons for vitamin D deficiency in the country. The following quotes describe their point of view:

*In the past, women were wearing hijab, yet they had fewer bone problems*.

(Participant #2, Female)

*I do not believe hijab can cause vitamin D deficiency, as women can go out at any time. It’s the hot weather that stops them*.

(Participant #8, Male)

### 3.4. Gender Differences Regarding Taking Actions against Vitamin D Deficiency

Many female participants, who were vitamin D-deficient, were keen to take actions to improve their vitamin D status. They admitted that doctors had advised them and suggested some methods to increase vitamin D status. These actions and methods included practicing activities to help increase sun exposure, the ingestion of vitamin D supplements, and the consumption of food rich in vitamin D. Some females’ quotes illustrate this:

After I gave birth to my little girl, I was exposing myself and my baby to sunlight to gain vitamin D. I also was taking vitamin D supplements, as I found that I was vitamin D deficient.

(Participant #1, Female)

*I was taking vitamin D supplements as the doctor asked me to do after I was diagnosed with vitamin D deficiency. Currently, I go to the gym three times a week to expose myself to sunlight and drink fortified milk. I also repeat the blood test every six months*.

(Participant #2, Female)

*I was diagnosed with vitamin D deficiency and currently take vitamin D supplements, as I only expose myself to sunlight through windows at my home*.

(Participant #4, Female)

On the other hand, only a few male participants said they did the blood test and measured serum vitamin D levels after the doctor advised them. One male stated that since he was told that he was vitamin D deficient, he did not take any actions to improve his vitamin D status, but he plans to take some supplements to improve his vitamin D status.

*Even though I work outdoors every day and expose myself to sunlight for long hours, I am vitamin D deficient and planning to take vitamin D supplements in the future*.

(Participant #22, Male)

The rest of the male participants did not do a blood test for vitamin D even though doctors had advised some of them to do it. Also, some of them said they were taking multivitamin supplements and drinking milk. They believed this was enough to get sufficient vitamin D levels. For example, one participant stated:

I take the required amount of vitamin D through drinking fortified milk with vitamin D and taking multivitamin supplements.

(Participant #13, Male)

## 4. Discussion

In the present study, we used a qualitative approach to explore knowledge and attitudes about vitamin D among adults in Saudi Arabia. We also explored social and cultural factors that might potentially contribute to vitamin D deficiency in the country. This study has revealed the following key findings: (1) Study participants showed a reasonable level of knowledge about vitamin D in areas related to the effect of vitamin D deficiency on bone health. Participants also had a general understanding of the importance of sun exposure as the main source of vitamin D and of the consumption of vitamin D supplements as an affordable source of this vitamin. However, there was limited knowledge regarding food rich in vitamin D; (2) The participants had mixed feelings toward sun exposure. Some of them had positive attitudes toward sun exposure and were willing to expose themselves to sunlight; however, it was based on some conditions, such as avoiding the high temperature during the middle of the day. Also, some participants expressed negative attitudes toward sun exposure and even avoided sunlight; (3) Participants who did not mind sun exposure were mostly exposing only their faces and hands to sunlight; (4) Several barriers for sun exposure identified by the participants are included hot weather, house designs and living in high-rise buildings, and limited public areas allowing outdoor activities that encourage exposure to sunlight; (5) Lifestyle issues such as physical inactivity and some cultural and religious concerns such as wearing hijab are also factors impeding Saudi people from accessing sufficient sunlight; (6) Female participants were more enthusiastic about taking actions to improve their vitamin D status in comparison to male participants.

The present study indicated that participants generally recognized the importance of exposure to sunlight and the intake of vitamin D supplements as good sources of vitamin D. It is noted that the study participants might have limited knowledge about the food sources of vitamin D, as very few participants mentioned milk and cheese as possible sources of vitamin D. Also, some participants thought eating a variety of fruits and vegetables would protect people against vitamin D deficiency, which reflects that some of the participants had received confusing information about this vitamin. The majority of study participants pointed out the effect of vitamin D on bone health. Participants were also aware of the major sources of vitamin D, including exposure to sunlight and the intake of vitamin D supplements. Although food sources of vitamin D were not commonly recognized among the participants, only 10–20% of vitamin D in human bodies is obtained through food sources [16]. Moreover, participants indicated their sources of information regarding vitamin D, including doctors, media, and colleges/universities. Hence, these are identified as valid channels to introduce and provide information about vitamin D and the importance of vitamin D to the general population. Several studies indicated associations between vitamin D deficiency and several chronic diseases, such as CHD and diabetes [7,8,9,17]. The results of the present study will be useful for developing vitamin D prevention strategies that consider more relevant communication channels and target specific aspects of vitamin D deficiency.

The study results indicated that some participants had positive attitudes toward sun exposure, while others had the opposite attitudes and were avoiding sunlight. Negative attitudes toward exposure to sunlight were also reported among different populations, such as China and Australia [11,15]. Exposure to sunlight is a key determinant that affects vitamin D status. The effectiveness of sun exposure depends on different elements, including the season, the time of day, duration of exposure, and body parts that are exposed to sunlight. In the current study, the constant hot temperature in the country was the main factor affecting study participants’ attitudes and behaviors toward sun exposure, as many participants reported that they avoid the strong heat, and it can cause them some health problems, such as allergic reactions and dizziness. The summer season can last for a long period, from March to November, in Saudi Arabia, and in the western region, in particular, it lasts almost all year round; thus, the majority of the participants considered hot climate as a key barrier to sunlight exposure in Saudi Arabia. Furthermore, besides the hot temperature, Saudi Arabia has unique cultural and religious principles of women wearing hijab to cover themselves. Also, for Saudi males, the traditional clothes have the same features of covering the body parts, but the only difference is in the color (males wear white thawbs, and females wear black abayas). Wearing clothes that cover most of the skin can affect the synthesis of vitamin D, as the skin is not exposed to UVB rays [18]. Thus, the cultural and religious practices, as well as the hot climate in the Kingdom, are considered to have a strong impact on receiving sufficient sun exposure among the population. Moreover, our results suggest that participants may not be completely aware of the amount of sun exposure body parts need to achieve sufficient vitamin D status. Most of the participants who did not mind sun exposure were exposing only their faces and hands to sunlight, and they considered themselves as people who like exposure to sunlight and probably believe it is sufficient. In Saudi Arabia, people usually have darker skin by nature, which affects vitamin D synthesis. The amount of melanin in the skin absorbs UVB which, as result, reduces vitamin D synthesis by slowing the conversion of 7-dehydrocholesterol to vitamin D3 in the skin [18]. Hence, people with darker skin have less efficient vitamin D production in the skin compared to people with lighter skin [18]. This, in fact, makes it more important for Saudi people to expose more parts of the body for a longer time to achieve sufficient vitamin D. The available evidence on sun exposure suggests spending 15 min exposed to sunlight without using sunscreen in order to get the beneficial effect of sunlight and avoid the damaging effects at the same time [19]. However, as we indicated previously, because Saudi people have darker skin and mostly wear clothes that cover their skin, they probably need one hour or more of sun exposure to produce the required amount of vitamin D. More research is needed to determine the required length of time of sun exposure to produce a sufficient amount of vitamin D in people with darker skin who cover their skin.

Our results also showed that living in modern buildings (such as high-rise buildings) due to the changes in house designs in the country is a reason preventing many people from getting exposed to sunlight, especially women. As mentioned previously, many female participants were living in units in high-rise buildings with no open areas, which make it difficult for them to expose themselves to sunlight. These results are consistent with a study conducted in Saudi Arabia that reported that the majority of women with vitamin D deficiency were living in units or small houses in crowded suburbs that do not get enough sunlight [20]. Similarly, a study conducted in Australia showed that living in high-rise buildings was linked to the high prevalence of vitamin D deficiency among migrants [21]. Also, some lifestyles factors, such as using cars for transportation and rarely walking outside as well as physical inactivity, lead to reduced opportunities for sunlight exposure. This also has a significant impact on public health in respect to the amount of physical activity required to prevent chronic diseases such as obesity and CHD.

The present study indicated that female participants were keener to take some actions to improve their vitamin D status than the male participants after they were diagnosed with vitamin D deficiency. Many female participants started consuming vitamin D supplements after consulting with their doctors, while only two males reported consuming multivitamin supplements. Females also intended to expose themselves to sunlight to increase their serum vitamin D levels. Several studies have shown gender differences in health-related behaviors, indicating that women are more health conscious than men [22,23]. A study in the USA reported that women aged 17–44 years were visiting physicians twice as much as men of the same age. Also, women were 50% more likely to obtain prescription medicine and use that medicine than males of the same age [24].

Based on our findings, there is a need for health education to increase the awareness about the importance of vitamin D and its health effects rather than focusing on only bone health. Furthermore, increasing awareness about the required duration of sun exposure, time of the day, and body parts that need sun exposure is very important in order for people to have sufficient sun exposure. In addition, increasing the general population’s awareness about the food sources of vitamin D and what fortified food is available should also be emphasized to prevent vitamin D deficiency. In Saudi Arabia, milk is fortified with vitamin D, including buttermilk, powder milk, and fresh milk [25]. Also, some kinds of cheese and breakfast cereals are fortified with vitamin D [25]. Moreover, there is a need to increase the availability of suitable places for outdoor activities such as walking and exercising for both genders and to provide private places for women only to allow them to uncover themselves and get the required sun exposure in a cultural appropriate manner. These places, such as gyms and health centers, should be available at affordable prices. Because of the hot climate in the country, increasing the awareness of the benefits of vitamin D supplements is very vital to reduce the high prevalence of vitamin D deficiency.

This study has a number of strengths. The interviews were conducted in the Arabic language, allowing direct communication with the study participants to gain direct insight into the issues embedded in a complex social and cultural context. In addition, the researcher who conducted the interviews (NA) is from the same ethnic group as the study participants and understands their culture and traditions, which helped participants to feel more trust and to speak up easily to share their experiences. Moreover, most existing studies addressing issues related to vitamin D deficiency used quantitative approaches. There is a shortage of qualitative knowledge, attitudes, and practice (KAP) studies that explore social and cultural factors which influence vitamin D deficiency in Saudi Arabia. This study has added further evidence to explore the underlying issues related to vitamin D deficiency in the Kingdom. The study also has some limitations. Since the interviews were conducted while patients were visiting hospitals, some patients were in a rush to leave to go back to work or to get their kids from school. In addition, due to the qualitative nature of this study, the results are not generalizable. Furthermore, as the participation in the study was based on the subject’s willingness to participate in the interview thus; it might introduce bias as subjects who agreed to be part of the interview might have better understanding of health knowledge than those who chose to not participate in the interview.

## 5. Conclusions

The current study revealed a number of significant findings that will provide health planners with an overview of the attitudes regarding exposure to sunlight among adults in Saudi Arabia and the barriers to adopting behaviors related to vitamin D. It also exemplifies the level of knowledge about different aspects of vitamin D in the country. Health education and promotion programs in the country should focus on the sources of vitamin D, including sun exposure, vitamin supplementation, and food sources, and should clarify the available food fortified with vitamin D in the Kingdom. They also need to increase the awareness about the long-term effect of vitamin D deficiency and its correlation with chronic disease. Health education programs should also highlight the importance of the consumption of vitamin D supplements as an affordable way to improve vitamin D status and should indicate the required amount of time of sunlight exposure in order to have sufficient vitamin D status. Moreover, health promotion efforts should also target males to increase their awareness of the importance of vitamin D and encourage them to adopt vitamin D-related behaviors that would be beneficial in improving vitamin D status. The government sector may take an important role in providing suitable areas for outdoor activities and sun exposure for both genders that would be culturally acceptable.

## Figures and Tables

**Table 1:** Socio-demographic characteristics of study participants.

Variables	Number	%
Group		
Cases	10	54.5
Controls	12	45.5
Age (years)		
<49	4	18
≥49	18	82
Gender		
Male	11	50
Female	11	50
Marital status		
Single	1	5
Married	17	77
Divorced	4	18
Citizenship		
Saudis	17	77
Non-Saudis	5	23
Place of residence		
Rural	0	0
Urban	21	95.5
Semi-rural	1	4.5
Education		
Up to primary levels	3	14
High School and bachelor or diploma degree	15	68
Master or PhD degree	4	18
Employment		
Employed (Full-time, Part time, self-employed)	15	68
Unemployed (Student, Retired, Housewife)	7	32
Family income (SR */monthly)		
<5000	6	27
5000–15,000	9	41
15,000–≥25,000	7	32

* Saudi Riyal 1 SR = 0.37 AUD.

## Supplementary Materials

The following are available online at www.mdpi.com/ 2227-9032/5/4/76/s1, Table S1: Questions Regarding Knowledge and Attitudes about Vitamin D, and Social and Cultural Factors that Contributes to Vitamin D Deficiency in Saudi Arabia.
