# Supplementary material for: Exploring Knowledge and Attitudes about Vitamin D among Adults in Saudi Arabia: A Qualitative Study
Source: Healthcare (Basel). 2017 Oct 16;5(4):76. doi: 10.3390/healthcare5040076 (PMC5746710; doi:10.3390/healthcare5040076)
Supplement: Supplementary file 1 [file healthcare-05-00076-s001.docx]

**Table S1.** Questions Regarding Knowledge and Attitudes about Vitamin D, and Social and Cultural Factors that Contributes to Vitamin D Deficiency in Saudi Arabia.

| **No.** | **Questions** |
| --- | --- |
| 1 | Can you tell me what do you know about vitamin D? Where did you hear about this? |
| 2 | Have you ever been offered any advice about preventing vitamin D deficiency? Who gave you this advice? Why was the advice given? |
| 3 | How do you feel about sun exposure? Do you like going into the sun? |
| 4 | Can you tell how often you are exposed to the sun? At what times of the day? |
| 5 | Which parts of your body do you usually expose to the sun? |
| 6 | What types of activities do allow you to expose to the sun adequately? Do you try to avoid these types of activities? |
| 7 | If you are rarely exposed to the sun, what do you do to avoid sun exposure? Can you please explain why? |
| 8 | In your opinion, what are the barriers to receiving adequate sun exposure? |
| 9 | Do you think that some cultural and religious factors may play a role in the high prevalence of vitamin D deficiency in Saudi Arabia? Can you please explain? |
| 10 | Have you heard of fortification? Can you explain what this is? |
| 11 | Do you take any vitamin D supplements? If yes, why are you taking them? |
| 12 | Can you tell me what the things you would do, to prevent vitamin D deficiency? |
